# Just how expert are “expert” video-game players? Assessing the experience and expertise of video-game players across “action” video-game genres

**DOI:** 10.3389/fpsyg.2013.00941

**Published:** 2013-12-16

**Authors:** Andrew J. Latham, Lucy L. M. Patston, Lynette J. Tippett

**Affiliations:** ^1^Department of Philosophy, The University of SydneySydney, NSW, Australia; ^2^Brain and Mind Research Institute, The University of SydneySydney, NSW, Australia; ^3^Department of Osteopathy, UNITEC Institute of TechnologyAuckland, New Zealand; ^4^School of Psychology, The University of AucklandAuckland, New Zealand; ^5^Centre for Brain Research, The University of AucklandAuckland, New Zealand

**Keywords:** video games, expertise, cognitive training, transfer of training, perceptual learning

Video-game play (particularly “action” video-games) holds exciting promise as an activity that may provide generalized enhancement to a wide range of perceptual and cognitive abilities (for review see Latham et al., 2013a). However, in this article we make the case that to assess accurately the effects of video-game play researchers must better characterize video-game experience and expertise. This requires a more precise and objective assessment of an individual's video-game history and skill level, and making finer distinctions between video-games that fall under the umbrella of “action” games. Failure to consider these factors may partly be responsible for mixed findings (see Boot et al., 2011).

## Assessing video-game experience and expertise

Current cross-sectional research investigating video-game play has relied on self-reports in order to distinguish expert video-game players (VGPs) from non-VGPs. Participants who report playing “action” video-games (e.g., Bialystok, 2006; Dye et al., 2009; Dye and Bavelier, 2010) for multiple hours per week, 6 months to a year prior to testing (e.g., Green and Bavelier, 2003; West et al., 2008; Hubert-Wallander et al., 2011) are classified as expert VGPs. Those who report no video-game play in the same period are classified as non-VGPs. Current criterion, however, fail to appreciate the significant difference between VGPs who have played for 5 h per week over the past 6 months and those who have played for 20+ h per week over the past 10 years (whom, in addition, would be classified as non-VGPs if currently abstaining from video-game play).

The purpose of cross-sectional research is to test the limits to which perceptual and cognitive processes may or may not be impacted by video-game play, while training studies using appropriate controls establish causal relationships between those differences and video-game play (see Boot et al., 2013). Unfortunately, the assumption that recent video-game experience reflects expertise is mistaken. There is no guarantee that VGP participants used in most current research papers possess either the experience or expertise necessary to be classified as expert VGPs. Similarly, there is no guarantee that individuals classified as non-VGPs, in their past, do not possess the relevant experience or expertise that would qualify them as expert VGPs. The misclassification of expert VGPs, non-VGPs or both, may be the basis of null results in the video-game literature (e.g., Murphy and Spencer, 2009; Irons et al., 2011), and other studies that have not been published.

A few early studies classified participants as expert VGPs and non-VGPs based on performance in a screening video-game (Greenfield et al., 1994; Sims and Mayer, 2002). As long as experimenters are able to set the appropriate performance threshold this is a valid method of classification. There is, however, a simpler method, used in other areas of expertise research (i.e., musical performance) that assigns expertise on the basis of professional attainment (i.e., highest instrument grade attained) and some objective assessment of their skill (i.e., achievement, awards or rankings). Similar measures of expertise are often freely available to video-game researchers on the internet and VGPs' in-game statistics. Level of professional attainment in video-game play can be assessed through placings in open tournaments and leagues, and qualifying, or being invited, to compete in closed tournaments and leagues. Like other competitions, video-game contests occur at a local, regional, national and international level, with each subsequent level representing higher levels of attainment.

Objective measures of video-game expertise are commonly available in the form of skill ratings and ladder rankings (based on the ELO system used in Chess) found in-game or online. For example, *Guild Wars 2* and *World of Warcraft* maintain ratings and rankings of individual players and teams. Some video-games do not assign exact ratings or rankings, but instead assign a token which represents skill level. For example, *Counter Strike: Global Offensive* assigns players one of 18 emblems ranging from Silver I to The Global Elite. Meanwhile, in *Starcraft II*, players are divided into different competitive tiers. The top 200 players on a server are in the Grand Master League, followed by the next 2% in the Master League and next 18% in the Platinum League. This is followed by Diamond, Gold, Silver, and Bronze, respectively. Finally, in some video-games, such as *Defense of the Ancients II*, ratings and rankings are maintained openly by online communities (e.g., *joinDota*, *GosuGamers*).

While video-game experience is not well suited to assigning expertise, it can highlight the qualitative and quantitative features of video-game engagement that may underlie expertise and its development. For example, Ericsson et al. (1993) used a diary study with musicians and found expert musicians engaged in more “deliberate practice” than non-experts. Deliberate practice refers to structured task rehearsal for the sake of improving performance, and is contrasted with “play” which is task immersion for the sole purpose of enjoyment. While many people play video-games, very few deliberately practice them. Engaging in deliberate practice is almost certainly also a characteristic feature of video-game expertise, however, video-games' success may come from an ability to blur the lines between deliberate practice and play.

Other relevant features of video-game experience include length of experience and the age at which they began gaming (e.g., Latham et al., 2013b). Unfortunately, potential variability in video-gaming histories increases the complexity of both variables. As a result, length of experience and age began might also be better understood in terms of play and deliberate practice. For example, a VGP may begin regular play during childhood, play more regularly and begin deliberate practice during adolescence, and then cut back to irregular play during tertiary study. Expertise-related changes are likely to reflect not just the accumulation of video-gaming experience but the nature of that experience as well, especially during formative years. The human brain is most malleable during childhood and adolescence (Freitas et al., 2011), thus perceptual, cognitive and neural changes resulting from intensive training (be it video-game, music or some other expertise) may be more likely during this period.

## Teasing apart major “action” video-game genres

Video-game researchers have largely restricted interest to the link between “action” video-game play and, perceptual and cognitive performance. The term “action,” however, actually refers to a vast array of different video-game genres. Early video-game researchers noted the significance of video-game type, showing that while spatially-orientated video-games enhanced visual cognition, non-spatially orientated games did not (e.g., Subrahmanyam and Greenfield, 1994; De Lisi and Wolford, 2002). Surprisingly, the importance of video-game genre has only recently been made apparent with real-time strategy (RTS) games shown to extend beyond the traditional results of enhanced visual cognition to improve higher order cognitive abilities, such as working memory and cognitive flexibility (e.g., Basak et al., 2008; Glass et al., 2013).

Briefly we highlight four major sub-genres that support international competition. These genres are: first-person shooters (FPSs), RTS, action RTS, and massively multiplayer online role-playing games (MMORPG). It is important to note that the complexity of these genres is greater than can be highlighted here (i.e., team roles, play-styles, meta-game), which may help shape specific perceptual and cognitive demands. In addition, there are many other “action” video-game sub-genres (i.e., driving, sport) with unique demands and potential to provide different sets of enhancements to players.

In RTS games players take control of a race, continually create, and utilize worker units to obtain resources, create, and expand a base, and create and improve combat units. Using combat units, players must destroy opponents or force them to concede. Countless combinations of build orders and unit combinations exist, which must be performed, controlled and adjusted in real-time against opponents. Success is reliant on the ability to assess, update and plan the most efficient series of mechanical responses. During professional *Starcraft II* play, players commonly execute up to 250 actions per minute, increasing to over 300 during combat. Other RTS games can have additional layers of complexity through the alternative victory conditions. For example, in *Civilization V* players can actively obtain victory through science, culture and diplomacy. Given these demands it is unsurprising RTS games may emphasize and enhance executive processes.

The term “action,” when used by researchers, however, has typically referred to FPSs. Players aim a targeting reticule at opponents and click in order to eliminate them. Success is dependent on the ability to make rapid visual judgments and responses. Although the executive demands are lower in FPSs than in RTS games, the demands on speed and accuracy of visual abilities are far higher. Many FPS games are objective and team-based (i.e., *Counter Strike: Global Offensive*) and include vehicles (i.e., *Battlefield 3*). However, even with these additions, success is still highly dependent on the speed and accuracy of basic visual and motor processes.

Action real-time strategy (ARTS) games arose from RTS games whereby players control a single unit with a handful of unique abilities called a “hero.” Often there are hundreds of unique heroes to choose from (e.g., *League of Legends* has 115 heroes and *Defense of the Ancients II* has 102). In a game, two teams of five players fight alongside waves of computer-controlled units in order to destroy the opponent base. Eliminating enemy heroes and units confers experience and currency. Experience allows heroes to gain levels which make them more powerful and grant skill points which are used to learn and improve skills. Currency is spent on items that either make a hero more powerful and provide additional skills, or supports the team by granting map vision, temporary invisibility, or revealing hidden units.

The competitive player-vs.-player element of many massively MMORPG shares some similarities with ARTS games. Players control a hero who has a whole pool of unique abilities to choose from rather than only a handful. Furthermore, “talent systems” allow players to customize their hero according to their specifications. However, unlike ARTS games, skills, talents, and items are selected prior to competing. With large pools of heroes, items, and abilities, the numerous possible combinations make each game played potentially unique. Success in ARTS and MMORPGs is reliant on ability to rapidly assess opponent hero roles and actions from visual cues. The specific perceptual and cognitive demands are roughly an intermediary between the RTS and FPS genres.

While there is undoubtedly a large overlap between the skills required to succeed across video-game genres (e.g., the ability to perform precisely timed bi-manual movements in response to complex visual cues), each genre typically has unique perceptual and cognitive demands necessary for success. Specific enhancements may result from these demands. Distinctions between genres are, therefore, of particular importance to researchers conducting training studies and those who wish to target specific abilities.

Researchers investigating expert VGPs typically provide lists indicating the “action” video-games participants report playing, with little appreciation given to the breadth of genres shown. Breadth itself may be another characteristic trait of experts, as the unique capabilities trained by specific tasks in a domain are likely to be advantageous to general performance within the domain as a whole. For expert VGPs, the unique capabilities trained by specific genres are likely to benefit video-game performance in general, and those with greater breadth may also tend to show greater expertise. As a result, the genre of video-games played needs to be considered in conjunction with video-game experience (see Assessing video-game experience and expertise).

Understanding the extent to which video-game play can shape perceptual and cognitive abilities requires testing expert VGPs. Current research, however, mistakenly classifies participants as expert VGPs using only a limited assessment of recent video-game experience, hindering progress in the field. While knowledge of a participant's video-game experience is incredibly useful, it cannot be used to definitively assign expertise. Proper classification of expertise requires the use of professional attainment, objective performance measures, or both. Once expertise has been correctly assigned, differences in experience between experts and non-experts may highlight factors, or combinations of factors, that promote the development and maintenance of expertise. Perhaps more significantly, it may reveal the key/s to shaping perceptual and cognitive processes.
